# The validity of central venous to arterial carbon dioxide difference to predict adequate fluid management during living donor liver transplantation. A prospective observational study

**DOI:** 10.1186/s12871-019-0776-9

**Published:** 2019-06-22

**Authors:** Mohamed ELAyashy, Hisham Hosny, Amr Hussein, Ahmed AbdelAal Ahmed Mahmoud, Ahmed Mukhtar, Amira El-Khateeb, Mohamed Wagih, Fawzia AboulFetouh, Amr Abdelaal, Hany Said, Mostafa Abdo

**Affiliations:** 1 Department of Anesthesia and Intensive Care, Kasr Al-Ainy Faculty of Medicine, Cairo University, Kasr Al-Ainy Street, Cairo, Egypt; 2 Department of Anaesthesia and Intensive care, Royal Brompton Hospital, RBHT, Sydney Street, London, SW3 6NP UK; 3 Department of Anesthesia and intensive care, Faculty of Medicine, Beni-Suef University, Beni-Suef, Egypt; 4 Department of Surgery, Ain Shams University, Cairo, Egypt

**Keywords:** Central CO2 gap, Pulmonary CO2 gap, Liver transplantation, Fluid resuscitation

## Abstract

**Background:**

To assess the validity of central and pulmonary veno-arterial CO_2_ gradients to predict fluid responsiveness and to guide fluid management during liver transplantation.

**Methods:**

In adult recipients (ASA III to IV) scheduled for liver transplantation, intraoperative fluid management was guided by pulse pressure variations (PPV). PPV of ≥15% (Fluid Responding Status-FRS) indicated fluid resuscitation with 250 ml albumin 5% boluses repeated as required to restore PPV to < 15% (Fluid non-Responding Status-FnRS). Simultaneous blood samples from central venous and pulmonary artery catheters (PAC) were sent to calculate central venous to arterial CO_2_ gap [C(v-a) CO2 gap] and pulmonary venous to arterial CO_2_ gap [Pulm(p-a) CO2 gap]. CO and lactate were also measured.

**Results:**

Sixty seven data points were recorded (20 FRS and 47 FnRS). The discriminative ability of central and pulmonary CO_2_ gaps between the two states (FRS and FnRS) was poor with AUC of ROC of 0.698 and 0.570 respectively. Central CO_2_ gap was significantly higher in FRS than FnRS (*P* = 0.016), with no difference in the pulmonary CO_2_ gap between both states. The central and Pulmonary CO_2_ gaps are weakly correlated to PPV [r = 0.291, (*P* = 0.017) and r = 0.367, (*P* = 0.002) respectively]. There was no correlation between both CO_2_ gaps and both CO and lactate.

**Conclusion:**

Central and the Pulmonary CO_2_ gaps cannot be used as valid tools to predict fluid responsiveness or to guide fluid management during liver transplantation. CO_2_ gaps also do not correlate well with the changes in PPV or CO.

**Trial registration:**

Clinicaltrials.gov Identifier: NCT03123172. Registered on 31-march-2017.

## Background

End-stage liver disease (ESLD) patients undergoing orthotopic liver transplantation can be prone to severe hemodynamic and metabolic changes. In the dissection phase; bleeding and hypovolemia are frequent [1], while in the an-hepatic period venous return may decrease resulting in a reduction in left ventricular preload [2] while after de-clamping and starting the neo-hepatic phase, the reperfusion injury and metabolic derangement can be severe enough to cause serious consequences [3].

Adequate tissue perfusion is an essential component of oxygenation during high-risk surgery and may improve the outcome [4, 5]. Proper monitoring of fluid resuscitation has been shown to reduce organ failure and hospital stay [6, 7]. The early warning signals of tissue hypoxia, such as lactate, central venous to arterial CO_2_ gradient and central venous oxygen saturation (ScvO_2_, 8], are essential indicators of the changes in the O_2_ delivery/consumption (DO_2_/VO_2_) relationship during high-risk surgery [8–10].

The difference between PCO_2_ in mixed venous blood (PvCO_2_) and PCO_2_ in arterial blood (PaCO_2_) is defined as the mixed venous-to-arterial CO_2_ tension gap [Pulm (P-a) CO_2_] and is affected by cardiac output and global CO_2_ production, as well as the complex relationship between PCO_2_ and CO_2_ content [11]. Normally, Pulm(P-a) CO_2_ does not exceed 6 mmHg. Elevated [Pulm(P-a) CO_2_] gradient has been observed in all types of circulatory failure (cardiogenic, obstructive, hypovolemic and distributive shock) [12].

Pulse Pressure Variation (PPV) is derived from the analysis of the arterial pulse waveform and is currently integrated in many monitors and is used as a valid tool to predict fluid responsiveness and to guide fluid management during liver transplantation [13].

To the best of our knowledge, no previous study assessed the ability of the Central CO_2_ gap or Pulmonary CO_2_ gap to predict fluid responsiveness and to guide optimization of fluid status during liver transplantation.

Our study aimed to assess the ability of the Central and Pulmonary CO_2_ gaps to guide adequate fluid management during liver transplantation. We hypothesize that CO_2_ gaps can be a complementary tool to PPV to guide adequate fluid management.

## Methods

This prospective observational study was approved by the Research Ethics Committee of Kasr Al-Ainy faculty of medicine, Cairo University (N-21-2017) and written informed consents was obtained from all study participants. The trial was registered prior to patient enrollment at clinicaltrials.gov (NCT03123172).

The study was designed to include 20 adult (> 18 years) ASA III to IV physical status patients with an end-stage liver disease (ESLD) scheduled for orthotopic liver transplantation. Patients were excluded if they were less than 18 years old or suffering from chronic respiratory disease. Induction of anesthesia was performed using propofol, fentanyl, and atracurium and maintained with sevoflurane adjusted to achieve an expired minimal alveolar concentration (MAC) between 1 and 2% in a mixture of air/oxygen, fentanyl infusion (1–2 μg/kg/h), and atracurium infusion (0.5 mg/kg/h). Patients were mechanically ventilated (Dräger Primus®, Germany) with a 6–8 ml/kg tidal volume and respiratory rate adjusted to maintain the ETCO_2_ between 4 and 4.6 kPa and positive end expiratory pressure (PEEP) of 5 cmH_2_O. Patients monitoring included five-lead ECG, pulse oximetry, invasive arterial blood pressure, core temperature, ETCO_2_, hourly UOP, and central venous pressure (CVP). A 7-Fr triple lumen CV catheter (Arrow International Inc., Reading, PA, USA) and an 8.5Fr pulmonary artery catheter sheath were placed in the right internal jugular vein and a pulmonary artery catheter (OPTIQ SVO_2_/CCO; Abbott Laboratories, North Chicago, IL, USA) was positioning guided by chamber pressures and confirmed with fluoroscopy. All patients received 6 ml/kg crystalloids as maintenance intraoperative fluid. Pulse pressure variations (PPV) [Philips Intellivue MP50 monitor (Philips Medical Systems, BG Eindhoven, The Netherlands)] used to guide intraoperative fluid management. If pulse pressure variation (PPV) was more than 15%, the patient was considered as a fluid responder and received a 250-ml boluses of 5% albumin to maintain ≤15% PPV Arterial, central venous and pulmonary artery blood samples were collected and analyzed (ABL 300, Radiometer Copenhagen, Denmark). We calculated the central venous to arterial CO_2_ gap [C(v-a) CO_2_] and the pulmonary mixed venous to arterial CO_2_ gap [Pulm(P-a) CO_2_] at two time periods, 30 min after the start of the pre-anhepatic dissection phase and 30 min after the reperfusion of the transplanted graft. No data was recorded during the an-hepatic phase or during partial or complete obstruction of the IVC by either clamping or surgical manipulation.

A transfusion trigger of 7 g/dL guided the need for blood transfusion while. Fresh frozen plasma and platelets were transfused if the INR reached > 1.5 and the count was < 50,000/μl respectively guided by thromboelastography and according to the severity of bleeding.

Patient characteristics; age, weight, MELD Score, child score and associated HCC were recorded. Intraoperatively central CO_2_ and pulmonary CO_2_ gaps were recorded apart from during the anhepatic phase and IVC obstruction as described earlier. Cardiac output (CO), lactate, central venous oxygen saturation (ScvO_2_) and PPV were all recorded throughout the procedures.

Primarily, the current study aimed to investigate the ability of CO_2_ gaps to predict fluid responsiveness appreciated by PPV. Area Under the Curve (AUC) for Receiver Operating Characteristic (ROC) was used to calculate the discriminative ability of both CO_2_ gaps to distinguish between FRS and FnRS with calculation of a cutoff value for either CO_2_ gaps should it be existing.

Secondarily*,* a comparison between central and pulmonary CO_2_ gaps in both fluid states (FRS and FnRS), the correlation of the CO_2_ gaps to the hemodynamic and metabolic parameters (PPV, CO and lactate), the correlation between hemodynamic and metabolic parameters (CO and lactate) and fluid responsiveness (FRS and FnRS) were also studied.

### Sample size calculation

The sample size was calculated after obtaining preliminary data of seven fluid non-responding status data points, which revealed a mean (SD) of the central CO_2_ gap to be3.8 (1.7). Assuming a mean difference of 30% between responding and non-responding and by using G power software (version 3.1.3, Heinrich-Heine-Universität, Düsseldorf Germany) with a power of 0.8 and 0.05 alpha error sample size was calculated to be 20 patients.

### Statistical analysis

Central and pulmonary CO_2_ gaps, cardiac output and lactate level are presented as mean (SD). Mann-Whitney test was performed for comparison of cardiac output and the Central and the Pulmonary CO_2_ gaps. The Receiver Operating Characteristic (ROC) curves were constructed, and the area under the curve (AUC) calculated to compare the performance of the central CO_2_ gap and the pulmonary CO_2_ gap in predicting fluid responsiveness. MedCalc version 12.1.4.0 (MedCalc Software bvba, Mariakerke, Belgium) generated values with the highest sensitivity and specificity (Youden index). Comparison of the AUC of the ROC curves used a Hanley-McNeil test. Correlations between either central CO_2_ gap and pulmonary CO_2_ gap and each of CO, lactate and PPV were done using Pearson moment correlation equation. A *P* value of less than 0.05 was considered statistically significant. All but ROC curves statistical calculations were done using SPSS (Statistical Package for the Social Science; SPSS Inc., Chicago, IL, USA) statistical program.

## Results

Twenty patients (16 males and 4 females) were enrolled in the study. Their mean (SD) age was 53.1(7.6) years, mean (SD) weight 79.2 (11.5) kg, and mean (SD) height 170.1 (7.2) cm. Thirteen patients had ESLD following hepatitis C, two patients had a hepatocellular carcinoma (HCC), and five patients had combined hepatitis C and HCC. Median (range) of MELD score was 17 (13–29). Fourteen patients had a child-class C, and six patients had a child-class B and fifteen patients presented with ascites. There were 67 data points recorded (20 FRS points and 47 FnRS points).

Mean values of central CO_2_ gap, pulmonary CO_2_ gap, lactate, ScvO_2_, and CO are presented in Table 1. Central CO_2_ gap was significantly higher in fluid-responder compared to the fluid non-responders (*P* = 0.016). Lactate level, ScvO_2,_ pulmonary CO_2_ and CO were comparable between both FRS and FnRS.

**Table 1:** Comparison between fluid responding status (FRS) and Fluid non-Responding Status (FnRS). Values presented as Mean (SD)

	Fluid Responding Status (*N* = 20)	Fluid non-Responding Status (*N* = 47)	*P* value
C(v-a) CO_2_gap	5.5(2.6)	4.3(3.2) *	0.016
Pulm(P-a) CO_2_ gap	5.16(4.24)	3.96(2.89)	0.18
Lactate	3.9(1.6)	3.5(2.3)	0.18
CO	6.7(2.6)	8.8(3.4)	0.06
Scvo_2_	80.3(12.1)	82.5(11.9)	0.32

*P value = 0.016 with significant difference between two groups. Mann Whitney test. *N* Number of data points, *C(v-a) CO2* central venous to arterial carbon dioxide tension difference, *Pulm(P-a) CO2* mixed venous to arterial carbon dioxide tension difference, *CO* cardiac output, *Scvo2* central venous oxygen saturation

**Fig. 1:**
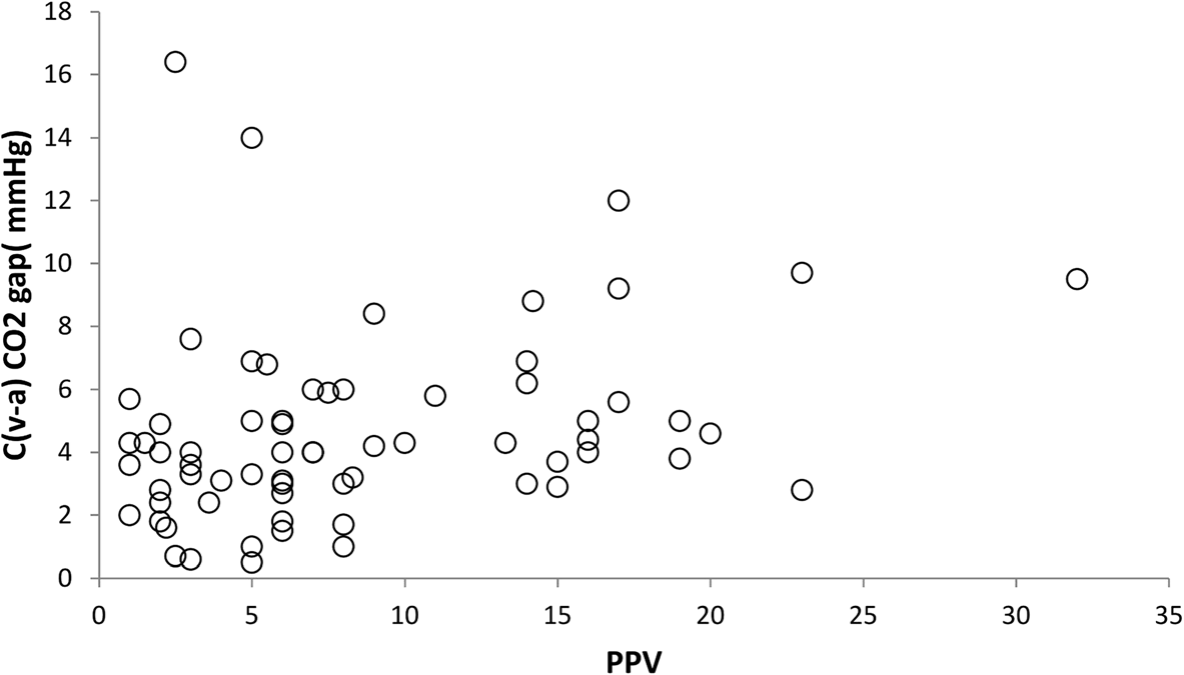
Correlation between PPV and C(v-a) CO2 gap. C(v-a) CO2; Central venous to arterial carbon dioxide tension difference, PPV; pulse pressure variation

**Fig. 2:**
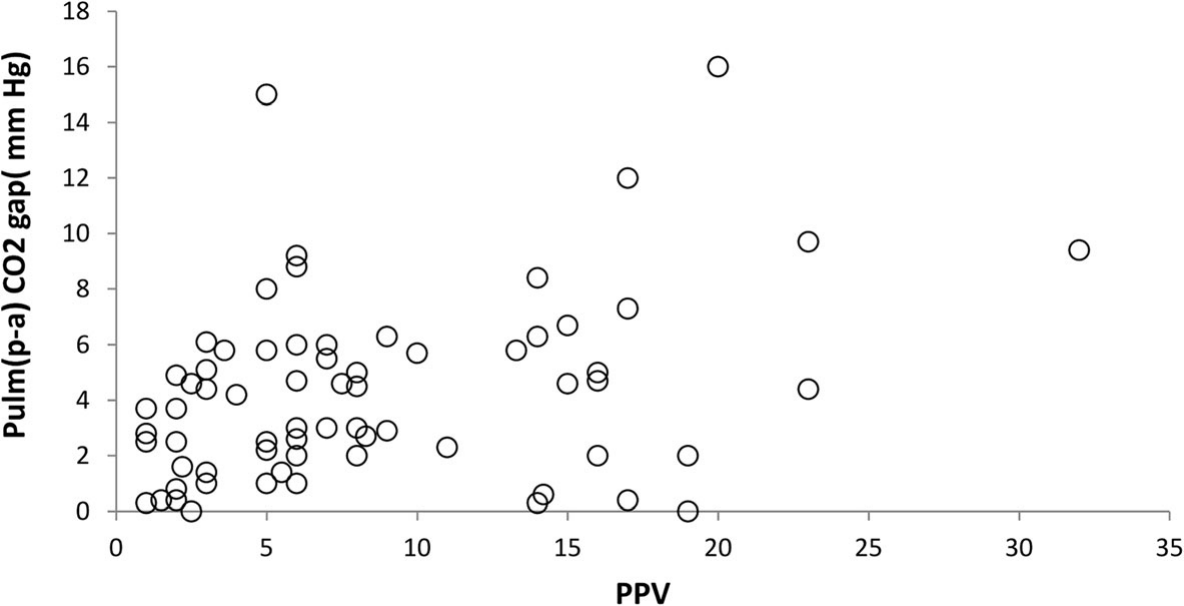
Correlation between PPV and Pulm(pv-a) CO2 gap. Pulm(p-a) CO2; mixed venous to arterial carbon dioxide tension difference, PPV; pulse pressure variation

A correlation was found between the central CO_2_ gap and PPV (r = 0.291, *P* = 0.017) (Fig. 1) and between the pulmonary CO_2_ gap and the PPV (r = 0.367 and *P* = 0.002) (Fig. 2).

**Fig. 3:**
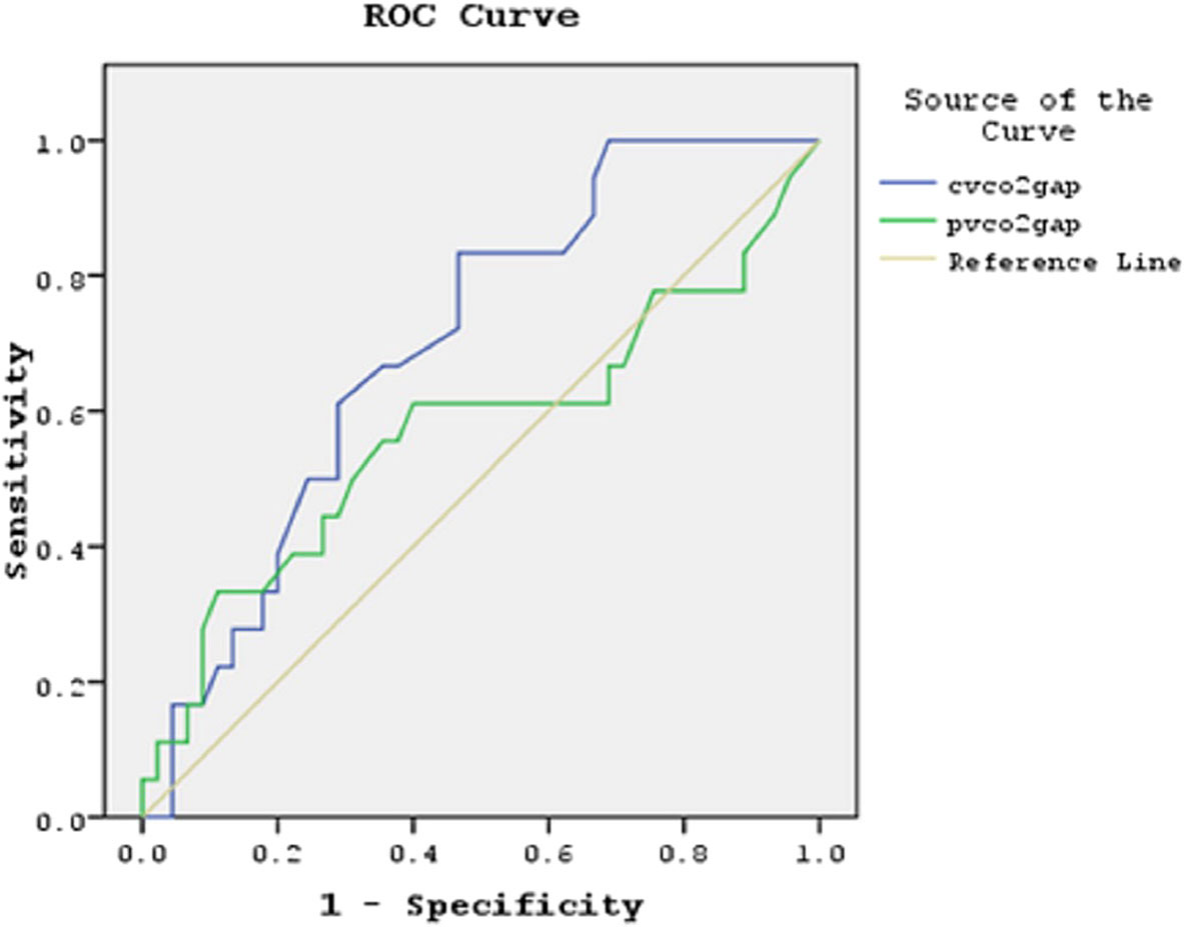
ROC curve of C(v-a) CO2 gap and Pulm(pv-a) CO2 gap. C(v-a) CO2; central venous to arterial carbon dioxide tension difference, Pulm(p-a) CO2; mixed venous to arterial carbon dioxide tension difference

The ROC for the central CO_2_ gap and pulmonary CO_2_ gap to predict fluid responsiveness was 0.698 and 0.570 respectively. From ROC curve, the optimal cutoff value 3.6 was determined for the central CO_2_ gap to predict fluid responsiveness with sensitivity 83% and specificity 55% (Fig. 3).

There was no correlation between central CO_2_ gap and CO (r = 0.168, *P* = 0.17) or between pulmonary CO_2_ gap and CO (r = 0.22) with *P* = 0.076. Also, there was no correlation between either central or pulmonary CO_2_ gap and the lactate level(r) = 0.071 and 0.202 respectively.

## Discussion

The target of the current study was to answer three questions; first, are the central and the pulmonary CO_2_ gaps valid indicators of fluid responsiveness in liver transplant patients? And is there a difference between the central and the pulmonary CO_2_ gaps in this setting? Second, do central and pulmonary CO_2_ gaps correlate with other hemodynamic and metabolic parameters such as CO, PPV and lactate? Third, are there any differences between fluid responding and fluid non-responding states in the hemodynamic and metabolic parameters?

For the first question, there were two main findings; (1) central CO_2_ gap was significantly higher in FRS than in FnRS during the pre- and post anhepatic phase of liver transplantation surgery, however the ability of the central CO_2_ gap to predict fluid responsiveness was weak (AUC = 0.698) and the cutoff gap value to predict fluid responsiveness was 3.6 mmHg. On the other hand, the pulmonary CO_2_ gap was comparable between FRS and FnRS. (2) Both central and pulmonary CO_2_ gaps were comparable (4.65 ± 2.996 versus 4.31 ± 3.34 respectively, *P* = 0.405) and both showed significant correlation (r = 0.444, *P* value = 0.0001). Possibly this contradiction between the two findings is the result of the presence of intrapulmonary shunt [14] in our patients characterized by cirrhosis and the high-risk present of hepato-pulmonary syndrome [15]. The similarity in hemodynamic pathophysiology between our patients and septic shock patients explains the agreement between our results and the previous findings of the use of CO_2_ gap in cases of septic shock, both gaps cannot be used alone as valid indicators of fluid responsiveness despite the central CO_2_ gap in our patients being higher in fluid responder, but the diagnostic validity of which remained weak. Based on our findings, veno-arterial CO_2_ gap cannot be relied upon as a tool to predict fluid responsiveness in these patients with complex hemodynamic and pathophysiological changes. Additionally, both CO_2_ gaps (central and pulmonary) are approximate and the central CO_2_ gap can replace the pulmonary [16–22].

Answering the second question*,* both CO_2_ gaps were only correlated with PPV but not with cardiac output or lactate level. PPV is a validated monitor for prediction of fluid responsiveness in major abdominal surgeries [13] however, the correlation of the CO_2_ gaps with PPV, despite being significant, was weak. This supports our finding that the CO_2_ gaps cannot be used alone as a valid predictors of fluid responsiveness in liver transplant patients.

Lactate level reflects both tissue anaerobic metabolism and the ability of the liver to metabolize it, with both conditions present in liver transplant patients during different phases of the transplant procedure (hepatic dissection, an-hepatic and neo-hepatic phases). Lactate level is a validated parameter to monitor adequate fluid resuscitation and the absent correlation between lactate and the CO_2_ gap in our patients supports the disputed validity of CO_2_ gaps as sole monitor of fluid responsiveness. Mekontso et al. [23] confirmed the correlation between CO_2_ gap and lactate level during hypoxic metabolic states with decreased oxygen consumption. Mekontso et al. used the ratio, rather than the absolute value, of CO_2_ gap to arterio-venous oxygen difference to relate to lactate levels.

For a constant total CO_2_ production (VCO_2_), changes in cardiac output result in large changes in pulmonaryCO_2_ gap at low cardiac output values, whereas changes in cardiac output will not result in significant changes in pulmonary CO_2_ gap at the high values of cardiac output [22, 24] This relation supports our finding of the absence of correlation between CO_2_ gaps and the CO in our patients known to have a high CO as part of the pathophysiology of liver cirrhosis.

Moving forward to the third question, FRS and FnRS patients were comparable regarding their lactate level_,_ pulmonary CO_2_ gap and CO. These findings support the verdict not to rely only on CO_2_ gaps alone as valid indicators of fluid responsiveness.

In our study, both central and pulmonary CO_2_ gaps correlated with PPV. Cuschieri et al. [25] and Van Beest PA et al. [26] showed strong agreement between central and pulmonary CO_2_ gaps in their studies of critically ill patients and on septic patients. In the current study, there was no correlation between central and pulmonary CO_2_ gaps with cardiac output., many studies [12, 25, 27] stated an increased central CO_2_ gap in low cardiac output states due to venous flow stasis which decreased with increased cardiac output. Cuschieri et al. [25] showed the correlation between the central CO_2_ gap and the pulmonary CO_2_ gap with cardiac index. Troskot et al. [12] concluded in their study of patients with severe sepsis and septic shock that the central CO_2_ gradient could predict fatal outcomes in non-ventilated patients only. Also, Mallat et al. [11] in their study on 80 patients with sepsis, measured the central CO_2_ gap and cardiac index using PICCO technology at time 0 (start of the study) and at time 6 (6 h after resuscitation) and found a correlation between CO_2_ gap and CI at T0 (r = − 0.69, *P* < 0.0001) and at T6 (r = − 0.54 *P* < 0.0001). Also, the changes in CI between T0 and T6 were also correlated with changes in CO_2_ gap (r = − 0.62, *P* < 0.0001).

In our study, the central CO_2_ gap did not correlate with cardiac output presumably due to the hyperdynamic state of the hepatic patient which preserves systemic blood flow even in states of tissue hypo-perfusion. Mecher et al. [28] studied 37 septic patients divided into two groups according to the central CO_2_ gap; high gap group > 6mmhg and normal gap group < 6 mmHg. They found normal gap group to have a high cardiac index (3 ± 0.2) despite circulatory failure. In this group; the gap did not change after fluid resuscitation (pre-fluid gap 4 ± 0 vs. post fluid 4 ± 1 mmHg) with an increase in cardiac index. While in the other group cardiac index was lower (2.3 ± 0.2) and gap decreased after resuscitation.

In our results, there was no correlation between either central CO_2_ gap or pulmonary CO_2_ gap and the lactate level. This was consistent with the study of Vallee et al. [29] in which 50 patients with septic shock, hyperlactatemia > 2 mmol/L and ScvO2 > 70% were enrolled. Patients were divided into two groups according to central CO_2_ gap with cut off value of 6 mmHg, low gap (< 6 mmHg), and high gap (> 6 mmHg). Patients’ resuscitation resulted in significantly larger clearance of lactate in low gap group than high gap group. There was also no correlation between CvCO_2_ gap and lactate level at time of inclusion T0 (r = 0.17, *P* = 0.22.) and poor correlation at six hours T6 (r = 0.37, *P* = 0.003) and twelve hours T12 (r = 0.36, *P* = 0.008).

In agreement with our results, Monnet et al. [30] found that volume expansion in all patients increased cardiac index and there was correlation between pulmonary CO_2_ gap and cardiac index at baseline (r = − 0.36, *p* = 0.0002) but not between pulmonary CO_2_ gap and lactate at baseline (*p* = 0.58). Also, Mecher et al. [28] showed no significant decrease in Pulmonary CO_2_ gap and lactate after fluid resuscitation in all patients with severe sepsis and systemic hypo-perfusion involved in the study.

fCO2 gap was found to be complementary tool for early resuscitation of patients with circulatory failure [31]. In the present study, despite the presence of significant difference in the central CO_2_ gap between fluid responding and non-responding states, the validity of CO2 gap is poor which makes its use to guide fluid resuscitation in liver transplant recipient is questionable.The present study had several limitations. First, This is a single center experience. Second, we avoided periods of marked hemodynamic instability caused by manipulation of the liver and downward retraction of the inferior vena cava which may intermittently obstruct venous return and causing hemodynamically significant changes in preload. Such changes in the preload are typically transient and may not reflect the actual volume status of the patient. Finally, we did not compare the CO_2_ gaps recorded during the pre-anhepatic phase to the CO_2_ gaps recorded during the neo-hepatic phase as the two periods represent different hemodynamic and pathophysiologic situations with the presence of a cirrhotic liver in the former and a potentially healthy graft in the latter. A future study can check this aspect.

## Conclusion

Both central CO_2_ gap and pulmonary CO_2_ gaps could not be used to predict fluid responsiveness or to guide adequate fluid management during living related liver transplantation. Both CO_2_ gaps could be used interchangeably, and both did not correlate well with changes in cardiac output or lactate level. These results suggest that CO_2_ gap may not be a good hemodynamic endpoint of resuscitation of patients undergoing living related liver transplant.

## Data Availability

The datasets used and/or analysed during the current study are available from the corresponding author on reasonable request.

## Abbreviations

ASA: American society of anesthesiologists
AUC: Area under the curve
C(v-a) CO_2_: Central CO_2_ gap
CO: Cardiac output
CO_2_: Carbon dioxide
CVP: Central venous pressure
DO_2_/VO_2_: O_2_ delivery/consumption
ECG: Electrocardiogram
ESLD: End-stage liver disease
ETCO_2_: End tidal CO2
FnRS: Fluid non-responsive status
FRS: Fluid responsive status
HCC: Hepatocellular carcinoma
MAC: minimal alveolar concentration
PAC: Pulmonary artery catheter
PaCO_2_: Arterial carbon dioxide pressure
PCO_2_: Partial carbon dioxide pressure
PEEP: Positive end expiratory pressure
PPV: Pulse pressure variations
Pulm (P-a) CO_2_: Mixed venous-to-arterial CO_2_ tension gap
PvCO_2_: Mixed venous carbon dioxide pressure
ROC: Receiver Operating Characteristic
ScvO_2_: Central venous oxygen saturation
UOP: Urine output
